# Synthesis of (*N,N'*-Bis(2-Hydroxyethyl)Ethane-1,2-Diamine)Malonatoplatinum(II) and X-Ray Crystal Structure of the *Cis-R,S*-Isomer

**DOI:** 10.1155/MBD.2000.349

**Published:** 2000

**Authors:** M. Galanski, M. Berger, B. K. Keppler

**Affiliations:** Institute of Inorganic Chemistry University of Vienna Waehringerstr. 42 Vienna A- 1090 Austria

## Abstract

Hydroxyethyl substituted amineplatinum(II) and (IV) complexes are an interesting class of platinum based antitumour compounds due to their uncoordinated hydroxy groups. These hydroxy groups could play an important role in the mode of action of such complexes with respect to their ability to act as donor or acceptor for hydrogen bonds. Moreover, their chemistry in solution is of interest because it was found that there is the possibility of an intramolecular attack to form ethanolatoamine chelated species which are responsible for very stable monoadducts with 5'-GMP. Furthermore, there is the possibility of derivatisation at the OH site to form a new series of platinum compounds which may be used for a carrier mediated transport to tumour tissues. In this context a series of (*N,N'*-bis(2-hydroxyethyl)ethane-l,2-diamine)-platinum(II) complexes has been synthesised. During purification of one of the platinum based compounds, it was possible to isolate (*SP*-4-3)-*R,S*-(*N,N'*-bis(2-hydroxyethyl)ethane-l,2-diamine)malonatoplatinum(ll) and to resolve the structure by single crystal structure analysis. Intra- and intermolecular hydrogen bonds have been found which may explain the spontaneous crystallisation of the *cis-R,S* isomer and the stabilisation of the boat conformation of the malonatoplatinum(II) six-membered ring.


### SYNTHESIS OF (*N,N'*-BIS(2-HYDROXYETHYL)ETHANE-1,2-DIAMINE)MALONATOPLATINUM(II) AND X-RAY CRYSTAL STRUCTURE OF THE *CIS-R,S*-ISOMER

M. Galanski, M. Berger and B. K. Keppler*

Institute of Inorganic Chemistry, University of Vienna, Waehringerstr. 42, A-1090 Vienna, Austria <keppler@ap.univie.ac.at>

Dedicated to the late Prof. Marc Leng

### ABSTRACT
Hydroxyethyl substituted amineplatinum(II) and (IV) complexes are an interesting class of platinum based antitumour compounds due to their uncoordinated hydroxy groups. These hydroxy groups could play an important role in the mode of action of such complexes with respect to their ability to act as donor or acceptor for hydrogen bonds. Moreover, their chemistry in solution is of interest because it was found that there is the possibility of an intramolecular attack to form ethanolatoamine chelated species which are responsible for very stable monoadducts with 5'-GMP. Furthermore, there is the possibility of derivatisation at the OH site to form a new series of platinum compounds which may be used for a carrier mediated transport to tumour tissues. In this context a series of (*N,N'*-bis(2-hydroxyethyl)ethane-1,2-diamine)-platinum(II) complexes has been synthesised. During purification of one of the platinum based compounds, it was possible to isolate (*SP*-4-3)-*R,S*-(*N,N'*-bis(2-hydroxyethyl)ethane-1,2-diamine)malonatoplatinum(II) and to resolve the structure by single crystal structure analysis. Intra- and intermolecular hydrogen bonds have been found which may explain the spontaneous crystallisation of the *cis-R,S* isomer and the stabilisation of the boat conformation of the malonatoplatinum(II) six-membered ring.

### INTRODUCTION
Since the discovery of cisplatin, $(NH_3)_2PtCl_2$[1,2], as anticancer drug and its subsequent worldwide clinical application for the treatment of different types of tumours[3], the synthesis and investigation of platinum compounds have become of interest. DNA is assumed to be the major target of a platinum based chemotherapy[4]. After DNA platination (clear preference for N7 of guanine) replication and transcription are inhibited and apoptosis is induced. The application of cisplatin in therapy is limited by a variety of side effects including dose dependent nephrotoxicity[5].
During the last decades, different strategies have been used to develop new platinum based anticancer compounds. Central points are reduction of toxicity and side effects, to increase the activity spectrum and to circumvent acquired cisplatin resistance of cells[6,7,8,9]. Therefore, thousands of platinum compounds have been synthesised and tested with respect to their anticancer activity. Nearly 30 entered clinical trials, but only carboplatin achieved worldwide approval and use. Based on a more detailed understanding of the mode of action of cisplatin and analogues, a rational drug design has started resulting in new platinum compounds and complexes which even violate the structure activity relationship: orally administrable platinum(IV) compounds, di- and trinuclear complexes, sterically hindered Pt coordination compounds, trans complexes and complexes with three nitrogen ligands.
In this context, we have synthesised mono- and bis(hydroxyethyl) substituted diamineplatinum(II/IV)[10,11,12] complexes which can be used for further derivatisation at the OH-group with respect to a carrier mediated transport of the cytotoxic moiety. Furthermore, the complexes themselves are very interesting because of their hydroxy-groups as a potential acceptor and/or donor for hydrogen bonds which could play an important role in the binding of platinum complexes to DNA. In addition, such complexes recently have shown an interesting behaviour in solution which resulted in very stable monoadducts with 5'-GMP[13,14].
During the synthesis of a series of (*N,N'*-bis(2-hydroxyethyl)ethane-1,2-diamine)platinum(II) complexes it was possible to isolate (*SP*-4-3)-*R,S*-(*N,N'*-bis(2-hydroxyethyl)ethane-1,2-diamine)malonatoplatinum(II) **1** and to resolve its structure by single crystal structure analysis.

### MATERIALS AND METHODS
### Chemicals
All chemicals obtained from commercial suppliers were used as received and were of analytical grade. Water was used bidistilled. The synthetic procedures were carried out in a light protected environment.

**NMR Spectra**

$^{1}$H-, $^{13}$C- and 2D NMR experiments were performed at 400.13 MHz ($^{1}$H) and 100.61 MHz ($^{13}$C) on a Bruker DPX 400 spectrometer (UltraShield™ Magnet) at 24°C. The gradient selected $^{1}$H,$^{1}$H- and $^{13}$C,$^{1}$H shift correlation experiments were performed using standard Bruker pulse programs.

**X-Ray Structure Determination**

The single crystal data were collected on a Nonius Kappa CCD diffractometer at room temperature. The measured intensities were corrected for Lorentz- and polarisation effects. The crystal structure was determined by direct methods (SHELXS-97, Sheldrick, 1997a)[15] and subsequent Fourier and difference Fourier syntheses. Final structure parameters of the compound were obtained by full-matrix least squares techniques on $F^2$ (SHELXL-97, Sheldrick, 1997b)[16]. X-ray structure analysis data are given in Table I. Crystallographic data for the structure reported in this paper have been deposited with the Cambridge Crystallographic Data Center as supplementary publication no. CCDC 155444[17].

**Table I.** X-Ray structure data of **1**.

| | |
|---|---|
| Molecular formula | $C_9H_{20}N_2O_7Pt$ |
| Formula weight (gmol$^{-1}$) | 463.36 |
| Space group | P2(1)/c |
| *a* (Å) | 9.086(2) |
| *b* (Å) | 9.267(2) |
| *c* (Å) | 15.960(3) |
| $\beta$ | 92.18(3) |
| *V* (Å$^3$) | 1342.9(5) |
| Formula units per cell | 4 |
| Density (gcm$^{-3}$) | 2.292 |
| Radiation (Mo K$\alpha$) (Å) | 0.71073 |
| $2\theta_{max}$ (°) | 28.26 |
| Reflns meas., Reflns unique, R(int) | 5780, 2987, 0.0220 |
| Independent data, $F_0>2\sigma(F_0)$ | 2751 |
| Variables | 181 |
| Largest difference peak and hole (e Å$^{-3}$) | 4.088/-4.868 |
| R1 [for $F_0>2\sigma(F_0)$] | 0.0534 |
| *wR*2 [for all $F_0^2$] | 0.1341 |
| GOF | 1.144 |

**Elemental Analyses**

Elemental analyses were performed by the microanalytical laboratory at the University of Vienna.

**Preparation of Platinum Complexes**

The synthesis of the title compound (*N,N'*-bis(2-hydroxyethyl)ethane-1,2-diamine)malonatoplatinum(II) (Scheme 1) starts from potassium tetrachloroplatinate(II) and *N,N'*-bis(2-hydroxyethyl)ethane-1,2-diamine. After activation of the resulting platinum(II) complex with silver sulfate, the aquasulfato species was then reacted with the in situ formed sodium salt of malonic acid. After addition of acetone and filtration the solution was left at 4°C to crystallize.

*Dichloro(N,N'-bis(2-hydroxyethyl)ethane-1,2-diamine)platinum(II).* A solution of *N,N'*-bis(2-hydroxyethyl)ethane-1,2-diamine (1.7916 g, 12.09 mmol) in 60 ml of ethanol was treated with $K_2PtCl_4$ (5.0176 g, 12.09 mmol). After addition of 115 ml of bidistilled water the pH was adjusted to 7 using diluted hydrochloric acid and stirred at room temperature. The precipitates were collected over a period of 20 hours. During this time the pH was kept constant at 7 using diluted NaOH. The yellow precipitates were washed with water and dried over $P_4O_{10}$. Anal. Calc. for $C_6H_{16}Cl_2N_2O_2Pt$: C, 17.40; H, 3.89; N, 6.76; Cl, 17.12; Pt, 47.10. Found: C, 17.45; H, 3.63; N, 6.62; Cl, 17.45; Pt, 47.23. Yield, 78.5%.

*Aqua(N,N'-bis(2-hydroxyethyl)ethane-1,2-diamine)sulfatoplatinum(II).* An aqueous suspension of dichloro-(*N,N'*-bis(2-hydroxyethyl)ethane-1,2-diamine)platinum(II) (2.7171 g, 6.56 mmol) was reacted with silver sulfate (1.9431 g, 6.23 mmol) at room temperature protected from light over night. The silver chloride precipitate was removed by filtration and the filtrate was evaporated to dryness under reduced pressure. This aqua species could be stored under nitrogen atmosphere over several days.

*(N,N'-Bis(2-hydroxyethyl)ethane-1,2-diamine)malonatoplatinum(II).* A solution of 0.1247 g of malonic acid (1.20 mmol) in 2.4 ml of 1.01 N NaOH was added to a solution of the aquasulfatoplatinum(II) complex (0.5483 g, 1.20 mmol) in 5 ml bidistilled water and stirred for 90 min. at 50°C and 16 hours at room temperature. After addition of acetone and filtration the solution was left at 4°C to crystallise. The crude product was dried over $P_4O_{10}$. Anal. Calc. for $C_9H_{18}N_2O_6Pt$: C, 24.27; H, 4.07; N, 6.29. Found: C, 23.28; H, 3.75; N, 5.92. Yield, 8.9%.

$^{1}$H NMR ($D_2O$): 2.5-3.1 (m, 8 H, H(a), H(b)), 3.49 (d, 1 H, $^{2}J_{H,H}$=15 Hz, H(d)), 3.55* (s, H(d)), 3.59 (d, 1 H, $^{2}J_{H,H}$=15 Hz, H(d)), 3.65-3.9 (m, 4 H, H(c), 6.34 (mb, 2 H, N*H*$_2$). $^{13}$C NMR ($D_2O$): 47.94 (1 C, C(d)), 48.00*, 53.96 (1 C, C(a)), 54.55*, 54.65*, 54.75 (2 C, C(b)), 57.67*, 57.99 (2 C, C(c)), 178.35*, 178.41 (2 C, C(e)), {chemical shifts marked with a * belong to another isomer}.

*(SP-4-3)-cis-R,S-(N,N'-Bis(2-hydroxyethyl)ethane-1,2-diamine)malonatoplatinum(II)* **1**. The crude product of (*N,N'*-Bis(2-hydroxyethyl)ethane-1,2-diamine)malonatoplatinum(II) (colourless crystals) was dissolved in water and left at 4°C for recrystallisation

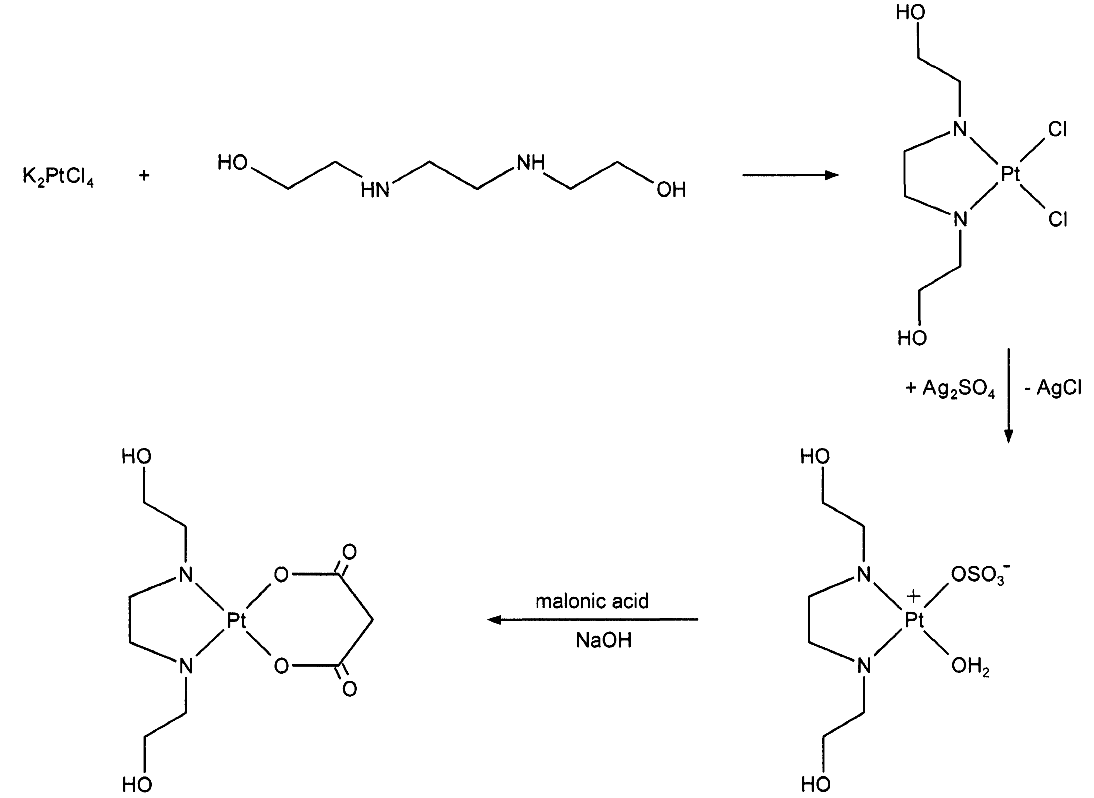

**Scheme 1.** Synthesis of (*N,N'*-bis(2-hydroxyethyl)ethane-1,2-diamine)malonatoplatinum(II).

**RESULTS AND DISCUSSION**

There is no chiral atom in the free diamine ligand *N,N'*-bis(2-hydroxyethyl)ethane-1,2-diamine. After coordination to the platinum(II) centre two chiral nitrogen atoms are produced resulting in the *trans-R,R-*, *trans-S,S-* and *cis-R,S* isomer[18]. Both trans forms are optical isomers (Figure 1).

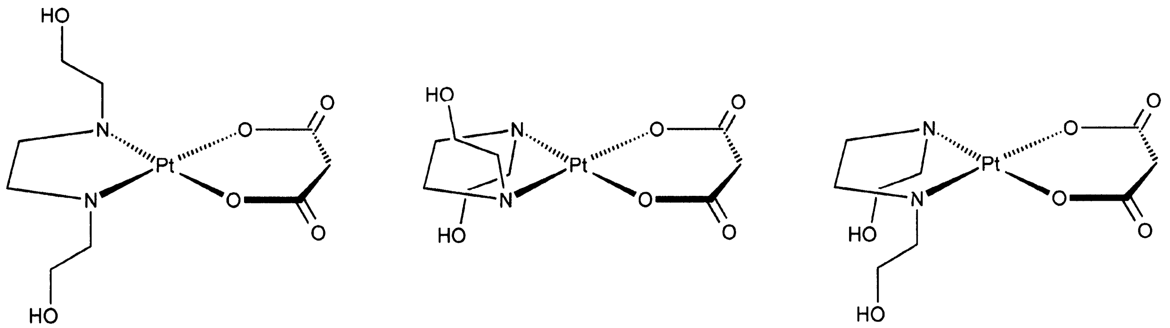

**Figure 1.** *trans-R,R-*, *trans-S,S-* and *cis-R,S* isomer of dichloro(*N,N'*-bis(2-hydroxyethyl)ethane-1,2-diamine)malonatoplatinum(II).

In the $^{1}H$- and $^{13}C$ NMR spectra of the crude product of (*N,N'*-bis(2-hydroxyethyl)ethane-1,2-diamine)malonatoplatinum(II) (Figure 2) which was obtained through addition of acetone to the reaction mixture, filtration and crystallisation at 4°C, more than one isomer could be detected

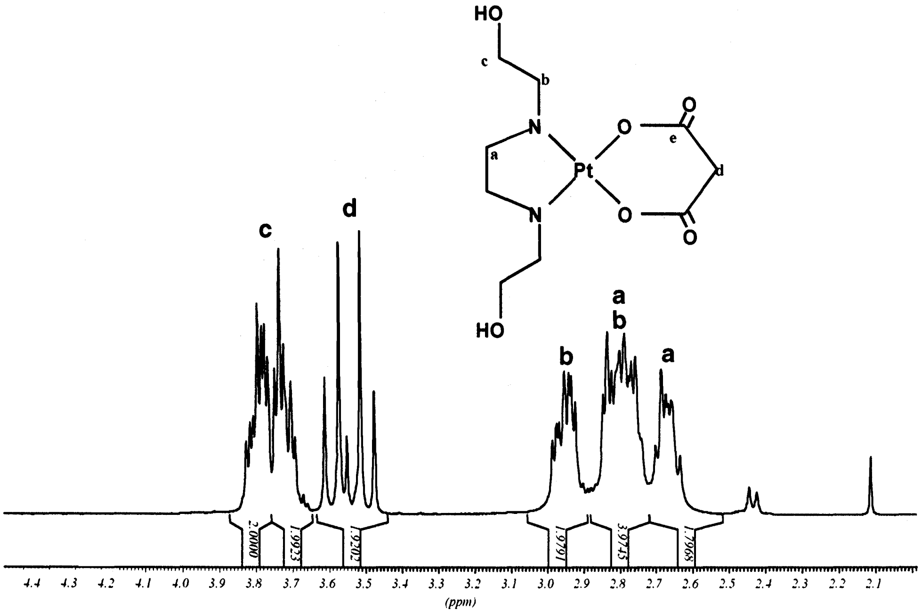

**Figure 2.** $^1H$ NMR spectrum of the crude product of (*N,N'*-bis(2-hydroxyethyl)ethane-1,2-diamine)-malonatoplatinum(II).

In the region between 2.5-3.1 and 3.65-3.9 ppm where the methylene protons H(a), H(b) and H(c) resonate, complicated multipletts can be observed which are not clearly resolved. In contrast, the H(d) protons between 3.45 and 3.65 ppm are isolated from the other resonances and can therefore be used to estimate the ratio of the isomers. Two dubletts for the non-equivalent diastereotopic H(d) protons of the *cis-R,S* isomer at 3.49 and 3.59 ppm with a $^2J_{H,H}$ coupling of 15 Hz (typical value for such geminal methylene protons) can be detected. A small singulett in the middle of the before mentioned dubletts at 3.55 which has no coupling to other protons in the $^1H,^1H$ shift correlation spectrum is also visible indicating the presence of one or both optical trans isomers (less than 10%).

In the $^{13}C$ NMR spectrum two sets of signals can be observed: 5 major resonances at 47.94 ( C(d)), 53.96 (C(a)), 54.75 (C(b)), 57.99 (C(c)), and 178.41 (C(e)) for the *cis-R,S* isomer and 5 small peaks at 48.00, 54.55, 54.65, 57.67 and 178.35 for one or both *trans* isomers.
After recrystallisation of the crude product at 4°C the *cis-R,S* isomer (*SP*-4-3)-*cis-R,S*-(*N,N'*-bis(2-hydroxy-ethyl)ethane-1,2-diamine)malonatoplatinum(II) **1** spontaneously crystallised (Figure 3) out of aqueous solution.

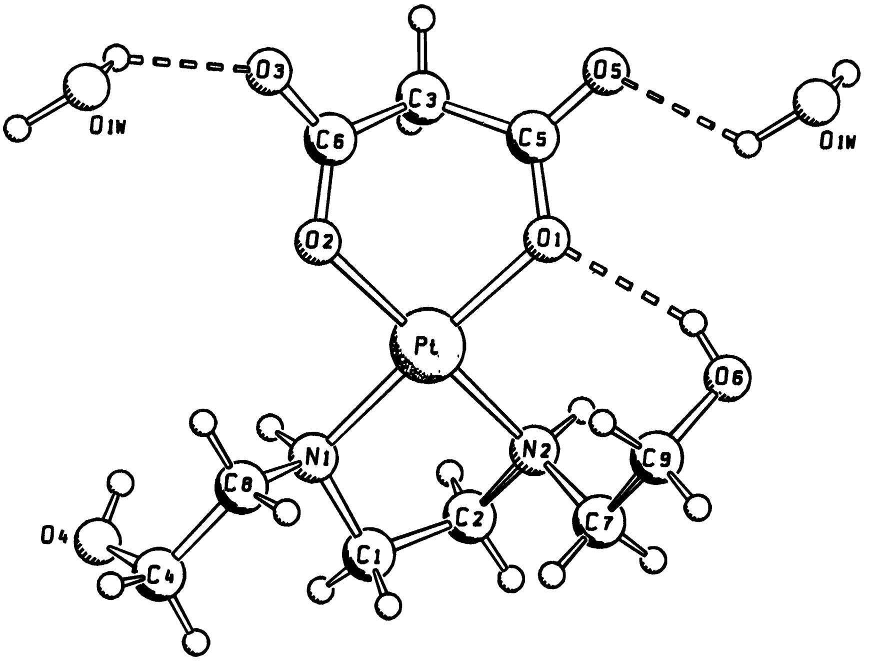

**Figure 3.** Structure of (*SP*-4-3)-*cis*-*R*,*S*-(*N*,*N'*-bis(2-hydroxyethyl)ethane-1,2-diamine)malonatoplatinum(II) **1** in the crystal. Selected distances (Å) and angles (°): Pt-O1 2.034(5), Pt-O2 2.035(5), Pt-N2 2.037(6), Pt-N1 2.044(6), O1-C5 1.289(8), O2-C6 1.286(8), O3-C6 1.231(8), O5-C5 1.220(8), C3-C5 1.510(10), C3-C6 1.529(10), C1-C2 1.515(11); O1-Pt-O2 89.4(2), O1-Pt-N2 91.9(3), O2-Pt-N1 93.4(3), N2-Pt-N1 85.4(3), O2-Pt-N2 178.24(18), O1-Pt-N1 177.24(19), C5-O1-Pt 120.6(4), C6-O2-Pt 120.0(4), C5-C3-C6 114.1(6), O3-C6-O2 122.8(6), O5-C5-O1 121.8(6), O5-C5-C3 119.9(6), O1-C5-C3 118.2(5), O3-C6-O2 122.8(6), O3-C6-C3 1.18.4(6), C1-N1-Pt 108.5(4), C2-N2-Pt 105.5(4), N2-C2-C1 107.7(5), N1-C1-C2 109.1(5).

The O and N atoms are arranged in a square planar manner around the platinum(II) centre. The angular sum is 360.1° with angles of 85.4(3)° N2-Pt-N1, 89.4(2)° O1-Pt-O2, 91.9(3)° O1-Pt-N2 and 93.4(3)° O1-Pt-N2, respectively. The angular sums in the ethane-1,2-diamineplatinum(II) chelate (five-membered ring) is 516.2°, whereas in the six-membered malonatoplatinum(II) ring it is 681.1°. The Pt-O and Pt-N distances (Pt-O1 2.034(5) Å, Pt-O2 2.035(5) Å, Pt-N1 2.044(6) Å, Pt-N2 2.037(6) Å) are in the normal range of aminecarboxylatoplatinum(II) complexes.

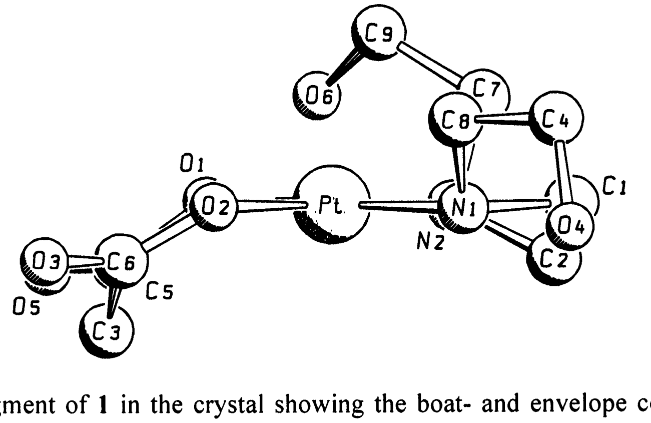

**Figure 4.** Structure fragment of **1** in the crystal showing the boat- and envelope conformation of the two platina rings.

The Pt-N1-C1-C2-N2 ring adopts an envelope conformation (Figure 4) with dihedral angles of 3.20, 30.50, 52.57, 47.60 and 24.48° at Pt-N1, N1-C1, C1-C2, C2-N2, and N2-Pt, respectively. In the Pt-O1-C5-C3-C6-O2 ring the dihedral angles at Pt-O1, O1-C5, C5-C3, C3-C6, C6-O2 and O2-Pt are found to be 42.25, 5.99, 50.52, 57.62, 5.20 and 35.67°. The values found are in good agreement with values expected for these conformations. In comparison, the dihedral angles in cyclopentane in the envelope conformation are 0, 25,

40, 40 and 25° (half chair conformation: 13, 34, 42, 34 and 13°) and in cyclohexane in the boat conformation 54, 0, 54, 54, 0 and 54° (chair conformation: 56°).

The structure of 1 is stabilised by one intramolecular and four intermolecular hydrogen bonds in the crystal (Figure 3 and 5).

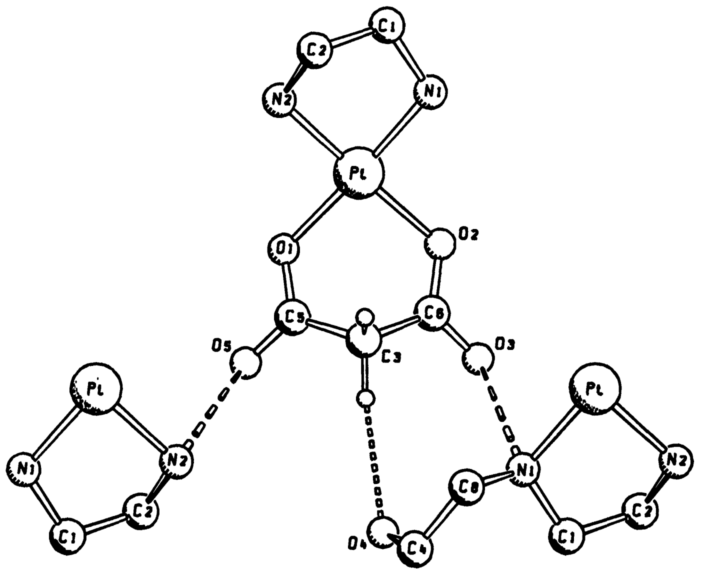

**Figure 5.** Structure fragment of **1** in the crystal showing the intermolecular donor acceptor contacts between N and O and the very weak C-H O interaction.

The intramolecular donor acceptor contact between O6 and O1 is 2.823(7) Å, (O6-H·····O1). Both oxygen atoms of the keto groups (O3 and O5) are involved in a hydrogen bond network to the water molecule in the crystal (O1W-O3, 2.742(7) Å, O1W-H·····O3 and O1W-O5, 2.831(7) Å, O1W-H·····O5) and to the nitrogen atoms N1 and N2 of the neighbouring ethane-1,2-diamine units (N1-O3, 3.033(8) Å, N1-H·····O3 and N2-O5, 2.904(8) Å, N2-H·····O5). These interactions seem on the one hand to stabilise the boat conformation of the malonatoplatinum(II) conformation. On the other hand the spontaneous crystallisation of the *cis-R,S* isomer could be explained by the intra- and intermolecular hydrogen bonds as possible driving force.

Moreover, a nearly linear arrangement of C3-H3·····O4 could be found with an angle of 172.17°. The distance between C3 and O4 is found to be 3.532(8) Å which is not in the range of C-H·····O hydrogen bonds (donor acceptor contacts around 3.2 Å) but may indicate a very weak attractive interaction further stabilising the structure of **1** found in the crystal.

### ACKNOWLEDGEMENTS

The support of the FWF (Fonds zur Foerderung der wissenschaftlichen Forschung) and COST (D8 program) is gratefully acknowledged. We thank Prof. G. Giester, Institute of Mineralogy and Crystallography, University of Vienna, for the crystallographic measurement and structural resolution and Mrs. R. Strobl for administrative support in the preparation of the manuscript.

### REFERENCES

[1] B. Rosenberg, *Inderdiscip. Sci. Rev.* 1978, **3(2)**, 134.
[2] B. Lippert (Ed.), Cisplatin: *Chemistry and Biochemistry of a Leading Anticancer Drug*, Weinheim: Wiley-VCH, 1999.
[3] M. C. Christian, *Semin. Oncol.* 1992, **19**, 720.
[4] E.R. Jamieson, S.J. Lippard, *Chem. Rev.* 1999, **99**, 2467.
[5] D. D. Von Hoff, R. Schilsky, Reichert. C. M., R. L. Reeddick, R. C. Young, F. M. Muggia, *Cancer Treat. Rep.* 1979, **63** 1527.
[6] L.R. Kelland, S.J. Clarke, M.J. McKeage *Plat. Met. Rev.* 1992, **36(4)**, 178.
[7] L.R. Kelland, *Crit. Rev. Oncol. Hematol.* 1993, **15**, 191.
[8] R.B. Weiss, M.C. Christian, *Drugs* 1993, **46(3)**, 360.
[9] E. Wong, C.M. Giandomenico, *Chem. Rev.* 1999, **99**, 2451.
[10] W. Zimmermann, M. Galanski, G. Giester B. K. Keppler, *Inorg. Chim. Acta* 1999, **292**, 127.

[11] W. Zimmermann, M. Galanski, B. K. Keppler, in Relevance of Tumor Models for Anticancer Drug Development, H. H. Fiebig, A. M. Burger (Eds.) in *Contrib. Oncol., W. Queißer, W. Scheithauer (Eds.)* Basel, Karger 1999, **54**, 447.
[12] M.Galanski, Ch. Baumgartner, M. Berger, W. Zimmerman, B. K. Keppler, in preparation.
[13] A. Zenker, M. Galanski, T. L. Bereuter, B. K. Keppler, W. Lindner, *J. Biol. Inorg. Chem.* 2000, **5**, 489.
[14] M.Galanski, W. Zimmerman, Ch. Baumgartner , B. K. Keppler, *Eur. J. of Inorg. Chem.*, in press.
[15] G. M. Sheldrick, *SHELXS-97,* Universityof Göttingen, 1997.
[16] G. M. Sheldrick, *SHELXL-97,* Universityof Göttingen, 1997.
[17] Crystallographic data (excluding structure factors) for the structure in this paper have been deposited with the Cambridge Crystallographic Data Center as supplementary publication no. CCDC 155444. Copies of the data can be obtained, free of charge, on application to CCDC, 12 Union Road, Cambridge CB2 1EZ, UK, (fax: +44 1223 336033 or e-mail: deposit@ccdc.cam.ac.uk).
[18] Q. Xu, A. R. Khokhar, J. L. Bear, *Inorg. Chim. Acta* 1999, **292**, 127.

**Received: February 21, 2001 – Accepted: February 28, 2001 –
Received in revised camera-ready format: March 1, 2001**

